# Implantable hyaluronic acid-deferoxamine conjugate prevents nonunions through stimulation of neovascularization

**DOI:** 10.1038/s41536-019-0072-9

**Published:** 2019-05-21

**Authors:** Alexis Donneys, Qiuhong Yang, Marcus Laird Forrest, Noah S. Nelson, Ti Zhang, Russell Ettinger, Kavitha Ranganathan, Alicia Snider, Sagar S. Deshpande, Mark S. Cohen, Steven R. Buchman

**Affiliations:** 1 Craniofacial Research Laboratory, Plastic Surgery Section, University of Michigan, Ann Arbor, MI 48109 USA; 2 Department of Pharmaceutical Chemistry, University of Kansas, Lawrence, KS 66047 USA; 3 Department of Surgery, University of Michigan, Ann Arbor, MI 48109 USA

**Keywords:** Fracture repair, Regenerative medicine, Translational research

## Abstract

Approximately 6.3 million fractures occur in the U.S. annually, with 5–10% resulting in debilitating nonunions. A major limitation to achieving successful bony union is impaired neovascularization. To augment fracture healing, we designed an implantable drug delivery technology containing the angiogenic stimulant, deferoxamine (DFO). DFO activates new blood vessel formation through iron chelation and upregulation of the HIF-1α pathway. However, due to its short half-life and rapid clearance, maintaining DFO at the callus site during peak fracture angiogenesis has remained challenging. To overcome these limitations, we composed an implantable formulation of DFO conjugated to hyaluronic acid (HA). This compound immobilizes DFO within the fracture callus throughout the angiogenic window, making it a high-capacity iron sponge that amplifies blood vessel formation and prevents nonunions. We investigated implanted HA-DFO’s capacity to facilitate fracture healing in the irradiated rat mandible, a model whereby nonunions routinely develop secondary to obliteration of vascularity. HA-DFO implantation significantly improved radiomorphometrics and metrics of biomechanical strength. In addition, HA-DFO treated mandibles exhibited a remarkable 91% bone union rate, representing a 3.5-fold improvement over non-treated/irradiated controls (20% bone union rate). Collectively, our work proposes a unique methodology for the targeted delivery of DFO to fracture sites in order to facilitate neovascularization. If these findings are successfully translated into clinical practice, millions of patients will benefit from the prevention of nonunions.

## Introduction

Delayed unions and nonunions are among the most debilitating fracture pathologies affecting approximately 250,000–500,000 patients per year.^1–6^ Angiogenesis and osteogenesis are intimately coupled, and impaired angiogenesis is often a predisposing factor underlying failed fracture healing. Vital temporal considerations exist in the relationship between nonunion development and angiogenesis. The first two weeks after fracture represent a critical window in the angiogenic-osteogenic coupling process whereby the effects of impaired neovascularization are intensified, potentially leading to nonunion.^7–15^ Conversely, this temporal niche may also represent a window of opportunity for targeting impaired angiogenesis therapeutically in order to prevent nonunions.

Emerging pre-clinical studies demonstrate utility in promoting angiogenesis during bone regeneration and healing utilizing therapeutic strategies. Perhaps the most promising is the use of deferoxamine (DFO) for these motives. DFO is a naturally occurring siderophore produced by *Streptomyces pilosus*, already FDA-approved for the treatment of transfusional iron-overload.^16^ Investigators have found an alternate utility for DFO as a potent prolyl-hydroxylase inhibitor (PHDi) and angiogenic stimulant. DFO triggers angiogenesis by chelating iron at the callus site and subsequently inhibiting the prolyl-hydroxylation of HIF-1α. In turn, this favors accumulation of HIF-1α, leads to nuclear translocation, dimerization with HIF-1ß, and transactivation of VEGF and other downstream mediators of new blood vessel formation.^17–20^ Utilizing this mechanism, DFO has been shown to augment angiogenesis in vitro and to augment callus neovascularization when serially injected into fracture sites in animal models, resulting in improved osteogenesis.^21–25^

Our laboratory has subsequently extended the use of this powerful strategy to improve pathologic fracture healing in a rodent model whereby the vascular microarchitecture has been altered and obliterated by radiotherapy, resulting in a high number of nonunions. The addition of serially injected DFO in these fractures elicited remediations in metrics of vascularity, osteocyte viability, callus mineralization and biomechanical strength. Moreover, these remediations facilitated a 2.35-fold improvement in bone union over non-treated/irradiated controls, in a model where nonunions are the expected outcome.^26–29^

Although these results are promising, the current method of DFO delivery in pre-clinical studies is impractical for human application and may impede its clinical translation. Due to its short plasma half-life, small size, rapid clearance and viscosity profile, multiple localized injections of DFO must be administered over a prolonged time-period, and accurate dosing is precluded by fracture site effusion. Furthermore, repeated injections introduce pain and inflammation, and increase the potential for infection at the healing interface.

To remove the need for repeated injections, we developed an implantable formulation to be introduced into the fracture site at the time of surgical repair. We controlled the timing of drug release by coupling DFO to a low molecular weight hyaluronic acid (HA) cross-linked scaffold, which provides structural support. HA is a linear hydrophilic polysaccharide, naturally occurring in the extracellular matrix of animal tissues.^30^ The HA carrier was selected based on its biocompatibility, biodegradability, and modifiable viscoelastic properties, which make it an attractive tissue filler device and vehicle for sustained drug delivery. While other researchers have reported modifications such as the use of liposomal formulations, dextrans and transdermal delivery systems to extend DFO release, HA exhibits additional features that make it more suitable in the setting of bone healing.^31–34^ HA has demonstrated intrinsic osteogenic capacity and anti-inflammatory properties that improve wound healing by minimizing tissue destruction secondary to inflammation.^35–38^ Additionally, HA has been FDA approved for decades as an injectable filler device, therefore its clinical utility and limited side-effect profile are well known.

Our sustained release kinetics were designed to facilitate DFO delivery over a 2–4-week period after implantation to ensure that the DFO was active before, during and after peak fracture angiogenesis which occurs approximately 7–14 days after bone injury. In addition, modifications in HA-DFO crosslinking were explored utilizing UV photo-crosslinking methacrylate chemistry. This enabled further extension in the retention time of the molecule.

The viscoelastic characteristics of HA-DFO facilitated localized implantation, maintained the drug at the fracture site for sustained release dosing of DFO over weeks, and eliminated the potential for fracture site effusion. Employing this technology in our established model of radiotherapy-induced non-unions, we investigated the therapeutic potential of HA-DFO to remediate obliterated vascularity and promote osteogenesis in the aftermath of radiation injury. Collectively, this work highlights a therapeutic strategy that may be readily translatable to offer a transformative solution for the management of clinical nonunions.

## Results

### Development of implantable HA-DFO

**Fig. 1:**
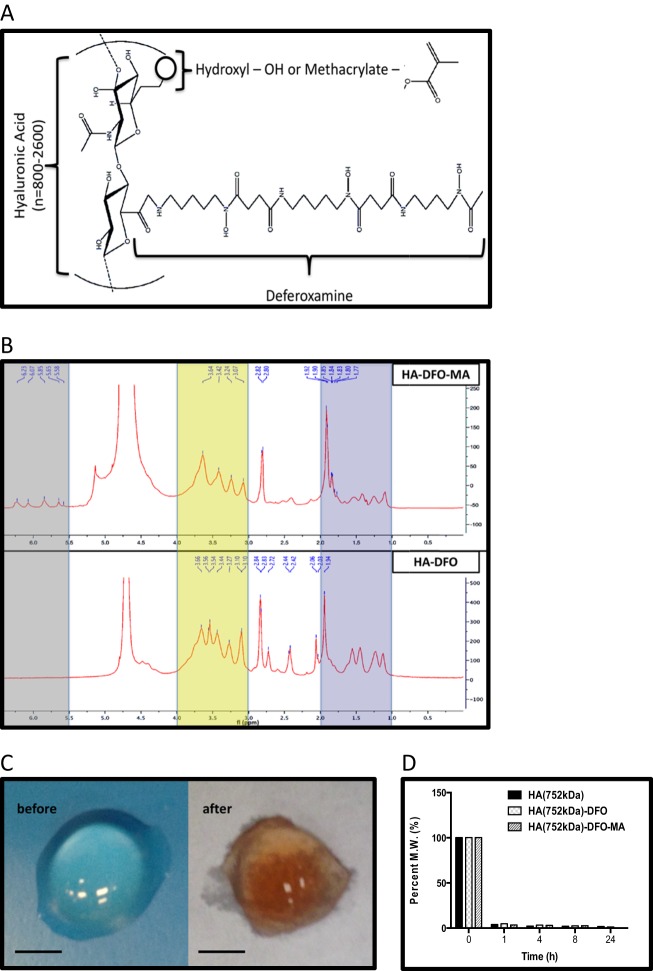
Development of implantable HA-DFO. **a** HA-DFO general chemical structure. **b** 1H-NMR spectra (D2O, 400 MHz) of HA (752 kDa)-DFO-MA and HA (752 kDa)-DFO confirming the characteristic peaks of MA (6.5–5.5 ppm), HA (4.0–3.0 ppm) and DFO (2.0–1.0 ppm), highlighted from left to right, respectively. The peak at *δ* = 4.70 in each spectrum corresponds to the solvent residue. **c** HA (752 kDa)-DFO-MA hydrogel before and after overnight incubation with iron. Note the rust color indicating successful iron chelation within the Hydrogel-Iron (III) complex. Scale bar length = 5 mm. **d** Percent molecular weight of unmodified HA (752 kDa), and its two derivatives in response to hyaluronidase enzymatic degradation

**Fig. 2:**
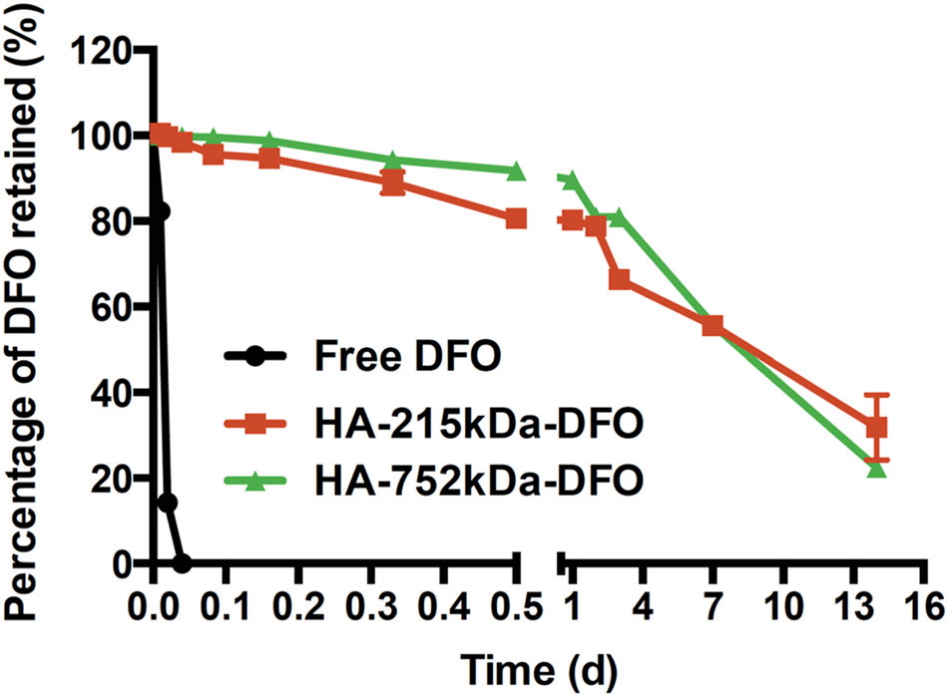
Release kinetics of free DFO and HA-DFO conjugates. In vitro testing of the three compounds in PBS at 37 °C (*n* = 3, Mean ± SD is shown). Note the rapid clearance of free DFO (*t*_1/2_~0.5 h) in comparison to the sustained release patterns exhibited by both HA-DFO conjugates (*t*_1/2_~10 d)

The general chemical structure of HA-DFO is depicted in (Fig. 1a). Various implantable formulations of HA-DFO differing in methacrylated crosslinking (See Figs 1b–d), or HA molar mass and DFO concentration (See Fig. 2) were synthesized and tested. These formulations were investigated utilizing (1) NMR spectroscopy, (2) spectrophotometric DFO quantification, (3) iron binding capacity, (4) enzymatic degradation, and (5) pharmacokinetic testing. The results of these five investigations are detailed as follows:

NMR spectroscopy demonstrated that the primary amine group of DFO was covalently conjugated to the unoccupied carboxylic acid groups of the HA-DFO backbone, and the successful conjugation was confirmed by the presence of characteristic peaks of Methacrylic anhydride (MA), DFO and HA in their ^1^H-NMR spectra (Fig. 1b). The degree of molar substitution was calculated to be approximately 64%, and the degree of weight substitution of the DFO to HA backbones was found to be 15.95%, indicating that nearly 90% of the conjugated DFO was maintained during the processes of reaction and purification.

Spectrophotometric quantification of DFO in the HA-DFO-MA conjugate demonstrated that 215–752 kDa conjugates retained 85–95% of the unmodified DFO’s binding capacity for Fe (III). When exposed to a UV source at 365 nm for 20 min in a 96-well plate, the clear solution turned into a clear and homogenous gel.

To observe iron binding capacity, HA (752 kDa)-DFO-MA was incubated with the FeCl_3_ solution overnight. The color of the hydrogel became orange-red, indicating formation of the Iron (III)-chelator complex (Fig. 1c).

To measure enzymatic degradation, the effects of hyaluronidase on the molecular weight of unmodified HA (752 kDa), and its two derivatives, HA (215 kDa)-DFO and HA (752 kDa)-DFO-MA were observed. The results are shown in Fig. 1d. The molecular weights decreased within 1 h to less than 5% of their initial molecular weights and to approximately 2% as the incubation proceeded. These results suggested that chemical modification of the HA on its carboxylic acid groups did not affect the hyaluronidase driven enzymatic degradation in simulated physiological environments.

The release profiles of free DFO and HA-DFO conjugates were evaluated in physiologic PBS solution at 37 °C. Release kinetics were fitted using a first-order model. As expected, free DFO exhibited a short half-life (*t*_1⁄2_ = 0.5 h). By comparison, both conjugates (HA-215kDa-DFO and HA-752kDa-DFO) demonstrated a sustained release pattern with 50% of the DFO being retained at 10 days (Fig. 2).

Ultimately, HA (752 kDa)-DFO (13% wt/wt) was selected for in vitro HUVEC applications and in vivo implantation in animal studies. The HA-752kDA-DFO formulation was chosen due to the similarity between release kinetics of the two formulations, as well as the fact that this formulation minimized reported concerns regarding DFO-induced cytotoxicity at high concentrations.^39^

### In vitro investigation of HA-DFO in irradiated HUVECs

**Fig. 3:**
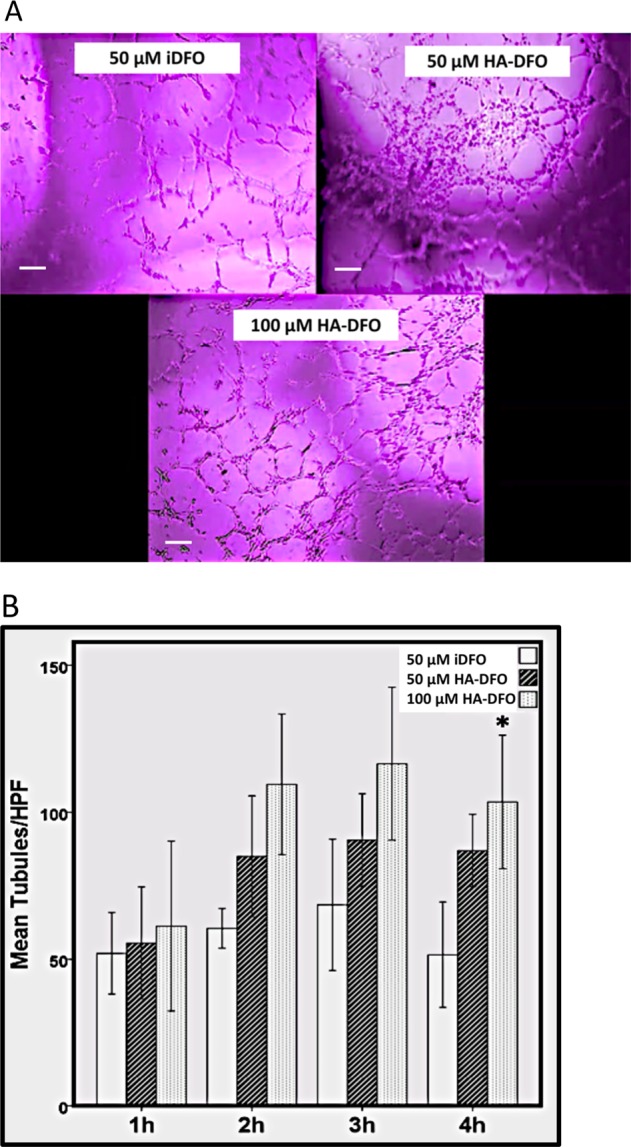
HA-DFO stimulates angiogenesis in vitro in HUVEC cells exposed to radiation. **a** Three groups of irradiated HUVECs demonstrate variable tubule formation in response to deferoxamine despite radiation injury. Scale bar length = 200 µm. Qualitatively, note the visibly increased tubule density and organization in the 100 µM HA-DFO sample when compared to 50 µM iDFO and 50 µM HA-DFO, even at only two hours of incubation (See Supplemental Material Movie S1 for recorded imaging). **b** Quantitatively, we observed a significant difference between 100 µM HA-DFO and both 50 µM doses of iDFO and HA-DFO at the 4-h time mark. Mean tubules per high power field are represented as group means and error bars indicate standard deviation. ANOVA was performed for group comparisons. All comparisons were performed within the respective timepoint. * indicates *p* < 0.05

Live cell imaging was performed in vitro to assess tubule formation as a proxy for angiogenesis. Based on our prior reported results, radiation significantly hampers tubule formation and leads to aggregation, clumping, and an inability of cellular organization, whereas injected DFO (iDFO) administration remediates these effects, leading to near normal tubule formation.^26,27^ Here, we examine the effects of HA-DFO in comparison to iDFO. Three groups of irradiated Human Umbilical Vein Endothelial Cells (HUVECs) were examined as follows: 50 µM iDFO, 50 µM HA-DFO, 100 µM HA-DFO. The experiment was conducted in triplicate. ANOVA was performed for group comparisons, and all comparisons were performed within the respective timepoint. *p* *<* 0.05 was considered statistically significant. We observed a significant increase between 50 µM iDFO and 100 µM HA-DFO at 4 h of incubation (*p* *=* 0.033) and trending increases between 50 µM iDFO and 100 µM HA-DFO at 2 and 3 h (*p* *=* 0.055 and 0.066), indicating superiority in tubule formation with 100 µM HA-DFO. Within 2 h of incubation, live cell imaging demonstrated more observable organization and more robust vascular networks with 100 µM HA-DFO when compared to other experimental groups. Though no statistical differences were observed between 50 µM HA-DFO and 100 µM HA-DFO at 2 and 3 h, the overall observed trend, in concurrence with a significant difference at 4 h, implied a dose-response relationship (Figs 3a, b). See Supplemental Material Movie S1 for recorded imaging.

### In vivo HA-DFO investigation in the irradiated non-union rat model

**Fig. 4:**
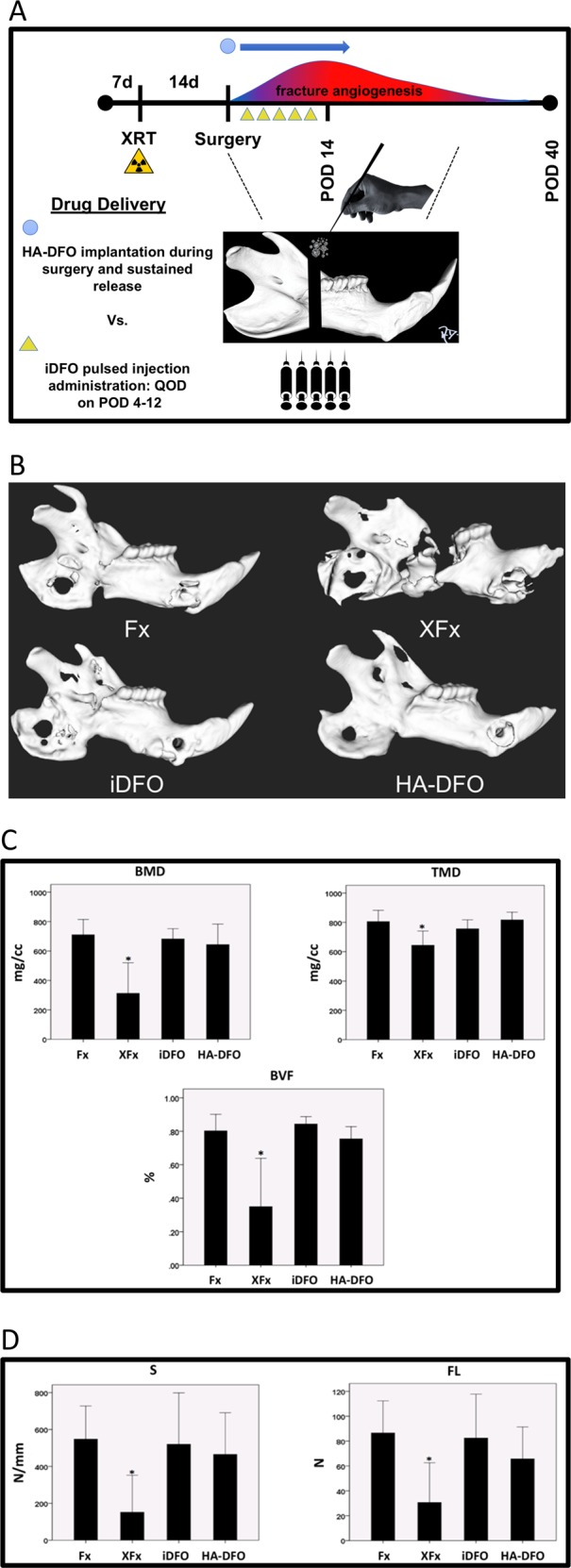
In vivo: HA-DFO restores mineralization and enhances biomechanical strength in irradiated fracture healing. **a** In vivo experimental timeline, normal peak fracture angiogenesis timing and schematic of the rat mandible depicting drug delivery methods and release kinetics. **b** Representative µCT images by treatment group. Notice the decreased bony bridging across the fracture site in the XFx sample that is restored in both iDFO and HA-DFO treated mandibles. **c** Radiomorphometrics: BMD, TMD, BVF, and (**d**) Biomechanical metrics: S and FL. ANOVA was performed for group comparisons. Group means are shown, and error bars indicate standard deviation. * indicates *p* < 0.05

Forty-four male Sprague Dawley rats underwent mandibular osteotomy and fixator placement immediately posterior to the third molar. Experimental animals received radiation (XRT) preoperatively, and received either no treatment, iDFO injection series postoperatively, or HA-DFO implantation intraoperatively. All animals underwent µCT analysis, biomechanical testing and bony union assessment (Fig. 4a).

Micro-CT (µCT) imaging demonstrated decreases in radiomorphometrics for the radiation group (XFx) that were remediated with the addition of both iDFO and HA-DFO therapies. For metrics of BMD, TMD and BVF, there was no difference between the two treatments, and both treatments improved upon XFx (Figs 4b, c). Biomechanical observations paralleled µCT findings. We observed decreases in Stress and Failure Load metrics for the radiation group that were restored with the addition of both iDFO and HA-DFO therapies. There was no difference in Stress and Failure Load between the two treatments, and both treatments were superior to XFx (Fig. 4d).

Bony union results demonstrated that non-irradiated fractures (Fx) reproducibly formed bony unions in 100% of cases, whereas only 20% of irradiated fractures (XFx) went on to achieve bony union. iDFO administration improved bony union to 67%, which represented an increase of 47-percentage points over XFx. Remarkably, we observed a 91% union rate in the HA-DFO group, representing an increase of 71-percentage points over XFx, and a 24-percentage point gain over standard iDFO (Table 1).

**Table 1:** Bony Union rates by treatment group

Fx	XFx	iDFO	HA-DFO
100%	20%	67%	91%

## Discussion

Delayed unions and nonunions are commonly caused by conditions such as underlying vascular disease resulting from diabetes, advanced age or uremia; pathologic states that directly weaken bone such as osteoporosis; anatomic predispositions to avascular necrosis; or iatrogenic causes associated with cancer management such as chemotherapy or radiotherapy. These conditions share a mechanistic commonality in that each impairs neovascularization during the fracture repair process.^7^ Here, we detail the composition of a novel implantable angiogenic/osteogenic stimulant, HA-DFO, and demonstrate its effective facilitation of fracture healing in an irradiated nonunion animal model. In vitro, results indicate that these effects are due to activation of angiogenesis, despite prior radiation-dependent inhibition of blood vessel formation. In vivo, our results suggest that implantable HA-DFO may be superior to iDFO in preventing nonunions.

The concept of augmenting vascularity to promote bone regeneration and healing has distinctive clinical origins dating back to the development of distraction osteogenesis. Gavriil Ilizarov discovered that mechanical tension introduced across a fracture site led to augmented vascularity and circulation above and beyond what would be expected during normal fracture healing. Reportedly, this hypervascular response treated osteomyelitis and nonunions at a time when antibiotics were not readily available.^40^ Since then, our knowledge and understanding of the angiogenic-osteogenic interface, and the timing of fracture callus vascularization, has increased considerably owing to the development of cellular, molecular and genetic methodologies. This increased understanding has created a renaissance of experimentation focused on identifying and implementing alternative triggers of angiogenesis to improve osteogenesis—as opposed to the mechanical stimulation techniques fostered by Ilizarov. The agents used in these studies vary, including the direct delivery of angiogenic growth factors, such as VEGF, FGF and TGF; regulation of angiogenesis inhibition with thrombospondin-2 modulation; less invasive mechanical stimulants such as ultrasound and alterations in weight bearing; and up-regulation of the HIF-1α pathway with DFO, L-mimosine and dimethyloxalylglycine.^7,41^ While non-angiogenic therapeutics such as recombinant human BMP-2 and 7, and Teriparatide have succeeded in translation, they have achieved only marginal clinical efficacy as osteogenic therapeutics due to associated complications and side effect profiles. Thus far, therapeutic angiogenic stimulants intended for enabling or accelerating osteogenesis have not yet reached clinical application.

With regards to the potential clinical translation of angiogenic stimulants, mediators of the HIF-1α pathway have offered the most persuasive evidence of clinical proximity and osteogenic efficacy. While the direct administration of VEGF is efficacious in preclinical models, the need for recombinant protein and/or genetic delivery approaches raise concerns that this technique is not yet ready for clinical adoption. This avenue is further encumbered by the high cost of producing such therapies.^22^ As such, Prolyl hydroxylase inhibitors, such as DFO, have emerged as a more attractive approach for promoting osteogenesis by indirectly stimulating angiogenesis through upregulation of the HIF-1α pathway. Wan and Shen et al. eloquently demonstrated the ability of DFO to accelerate bone regeneration during distraction osteogenesis, and femur fracture healing in murine models, respectively.^23,24^ Our laboratory subsequently investigated the ability of DFO to restore angiogenesis and fracture healing in a more rigorous animal model of irradiated nonunions, whereby the vascular microarchitecture was obliterated by a human-equivalent dose of radiotherapy. Collectively, we saw an improvement in the prevention of non-unions by 45 percentage points over non-treated controls in a model where nonunions are the expected outcome.^27,28^

Based on this work, the first in-human application of DFO to facilitate osteogenesis in previously irradiated bone was realized. In a collaboration between our laboratory, and reconstructive surgeons at Stanford University, the clinical repurposing, volumetric dosing and delivery of DFO to facilitate distraction osteogenesis in a young adult patient with maxillary hypoplasia secondary to previous radiotherapy was achieved.^42^ The clinical utility of distraction osteogenesis in patients exposed to radiation is currently eschewed due to the prohibitive incidence of nonunions. Nonunion in this setting is thought to result from the paucity of vascular ingrowth provided by irradiated tissues and the unmet high angiogenic metabolic demands incurred by mechanical bone regeneration. In this unique case, the patient underwent trans-cutaneous catheter placement to the right and left pterygo-maxillary regenerative sites to enable delivery of DFO prior to distraction. Our dosing was calculated volumetrically, based on the anticipated 3-D area of bone regeneration, and our experience afforded by our pre-clinical dosing of injectable DFO. Accidental dislodgement of the left catheter prior to distraction resulted in exclusive delivery of DFO to the right regenerate area, establishing the patient as his own control. We observed an increased bone area and radiodensity in the treated areas indicating accelerated bone regeneration due to the successful delivery of DFO at 3-months of consolidation (~25 and 30%, respectively). This case report demonstrates the clinical utility of this highly effective angiogenic/osteogenic therapeutic and demonstrates proof of concept for human application. Despite this promising result, this report also highlights the current cumbersome delivery methodologies, and timely need for more practical, implantable options for this efficacious therapy in order to facilitate its transfer from the bench to the bedside. A five-injection series, or catheter-based delivery system may be viewed as an undesirable last-resort option by patients, rather than a new standard of care. Although these cumbersome delivery methods ensure drug delivery into the osteogenic site, they would require frequent office visits or longer inpatient stays, both of which would carry substantial expenses and introduce the potential for human error. In addition, repeated injections or transcutaneous catheter delivery also expose the bony regenerate to skin flora, potentiating the risk of infection and failure of the reconstruction. Taken together, these numerous barriers make a fully-implantable DFO-construction an exceedingly attractive alternative.

Given these challenges, we have developed a novel HA-DFO conjugate to deliver DFO gradually to a fracture site over the length of the critical period of angiogenesis that is needed for new bone development. HA was first approved as a device in the 1960s and has since been applied as a filler cosmetically and for other structural injections or implantations in the body. It has gained popularity as a drug delivery agent only recently and has been shown to enhance locoregional drug retention along with substrate delivery of agents conjugated to its crosslinked backbone. This delivery platform can release a conjugated drug locally in soft tissue or deep tissue locations over a period of days to weeks depending on the avidity of the conjugation link or the degree of the HA polymer crosslinking.

The sustained release delivery of DFO through conjugation with HA allows for better drug-fracture interface during the critical angiogenic period of healing, allowing for superior union formation over pulsed DFO injections, without overgrowth of bone or biomechanical inferiority to normal fracture callus, even in the setting of high dose radiation damage. Another significant benefit of this technology is related to the kinetics of the HA-DFO release reaction, which limits free DFO concentrations to a fraction of what would be experienced through systemic delivery or repeated local injections of free DFO. The blood serum concentration of free DFO observed with HA delivery is therefore several orders of magnitude lower than the FDA-approved doses given for iron-overload treatments. Lastly, it is important to note that we did not expect improved bone union with HA-DFO, beyond what was observed with pulsed DFO injections. Rather, our intent was to investigate efficacy. While the iron chelating properties and angiogenic mechanisms of HA-DFO were confirmed in our in vitro studies, these new findings raise inquiries regarding the contribution of HA alone to the angiogenic and osteogenic mechanisms during irradiated fracture healing that will require further dedicated investigation. Taken together, these remarkable observations support the impact and potential of HA-DFO for preventing or treating delayed unions or nonunions. Advancement of this platform clinically could address a critical gap in the management of these challenging bone pathologies for patients who lack effective reconstructive options.

## Methods

Hyaluronan sodium salts (752 kDa) were purchased from Lifecore Biomedical, Inc. (Chaska, MN). Hyaluronic Acid (752 kDa)-Deferoxamine conjugate synthesized in house. Methacrylic anhydride, 2-Hydroxy-4′-(2-hydroxyethoxy)-2-methylpropiophenone (irgacure® 2959) and hyaluronidase from bovine testes (EC 3.2.1.35, 750–3000 U/mg) were purchased from Sigma Aldrich (St. Louis, MO). Sodium hydroxide was purchased from J.T. Baker (Center Valley, PA). Iron (III) chloride (anhydrous, 98%) was obtained from Alfa Aesar (Ward Hill, MA). Thermo Scientific™ SnakeSkin™ Dialysis Tubing (10 K MWCO) and organic solvents of analytical grade were obtained from Fisher Scientific (Lenexa, KS). Double distilled water (ddH_2_O) was used in syntheses, characterization and cell-culture (sterilized by autoclaving).

### Preparation of implantable HA-DFO conjugates

HA-DFO is produced by conjugating HA (0.2–1MDa) and DFO (10–15% w/w) utilizing [1-Ethyl-3-(3-dimethylaminopropyl)-carbodiimide] (EDC). EDC is a zero-length crosslinking agent used to couple carboxyl or phosphate groups to primary amines. The chemical modification of HA can be performed utilizing this method to conjugate DFO at its carboxyl region and forms a prodrug that is later cleaved at its target site. Methacrylated HA-DFO (HA-DFO-MA) was synthesized in order to facilitate crosslinking and further modify the degradation profile. MA was first attached to the carboxylic acid groups of the HA-DFO (752 kDa)-DFO conjugate following a procedure reported elsewhere with some modifications.^43^ Briefly, approximately 100 mg of the HA-DFO (17.8%, w/w) was dissolved in 7-mL ddH_2_O and then one molar equivalent of methacrylic anhydride was added in the solution with gentle stirring. The pH of the mixture was kept at 8 using a 2.5-N sodium hydride. The reaction was carried out at room temperature for 2 h, followed by storing at 2–8 °C overnight. Afterwards, the mixture was transferred into the dialysis tubing and dialyzed against ddH_2_O for 3 days. The dry form of the HA-DFO-MA was obtained via lyophilization. A stock solution of 10% w/v of Irgacure 2959 was freshly prepared in an ethanol-acetone mixture (70/30; v/v). Two microliters of Irgacure 2959 was added into 1 mL of HA-DFO-MA (10 mg/mL) in ddH_2_O at a concentration of 10-mg/mL, and the mixture was gently vortexed for 30 s. Approximately a 100-μL aliquot was transferred into the 96-well cell culture plate, which was subsequently exposed to UV lamp at a wavelength of 365 nm for 20 minutes.

### ^1^H Nuclear magnetic resonance (NMR) spectroscopy

The HA (752 kDa)-DFO or HA (752 kDa)-DFO-MA was dissolved in deuterium oxide (D_2_O) and their ^1^H NMR spectra were collected on a Bruker Avance 400 MHz NMR Spectrometer (Bruker Corporation, MA, USA).

### Spectrophotometric DFO quantification

The quantification of DFO in the HA-DFO-MA conjugate was determined spectrophotometrically by converting the methacrylated HA-DFO to the iron-saturated ferrioxamine using a modified method.^44^ Three mM of iron (III) chloride (FeCl_3_) was incubated with a series of standard solutions of DFO (0.05–0.5 mg/mL), 1-mg/mL HA (215 kDa or 752 kDa)-DFO-MA at ambient temperature overnight, followed by recording the absorption at 430 nm using a UV microplate reader (SpectraMax Gemini; Molecular Devices, Sunnyvale, CA). The amount of DFO in the formulation was determined by quantitating respective HA-DFO-MA generated ferrioxamine species with the calibration curve of the ferrioxamine standard solutions prepared from unmodified DFO.

### Iron binding capacity

Approximately 100 μL of HA (752 kDa)-DFO-MA was incubated with 500 μL 3-mM FeSO_4_ in a 1.5-mL Eppendorf tube, and the mixture was shaken overnight on an orbital shaker at 50 rpm overnight. The preparation was photographed before and after iron incubation to observe oxidation.

### Enzymatic degradation

The kinetics of enzymatic degradation of HA was studied by monitoring the molecular weight change. First, 1 mg/mL of unmodified HA (752 kDa), HA (752 kDa)-DFO or methacrylated HA (752 kDa)-DFO solutions was prepared in 4 mL of PBS (10 mM, pH7.4), and then hyaluronidase solution (10 μg/mL) was added into the reaction system. While the mixture was incubated at 37 °C, the molecular weights of 100-μL aliquots of the samples were monitored by gel-permeation chromatography (GPC) at 25 °C on a Shimadzu 2010CHT with a refractive index detector (RID-10A, Shimadzu Scientific Instruments, KS), using a Shodex OHpak SB-806 HQ column (Showa Denko America, Inc., New York, NY) with 5-mM ammonium acetate as the mobile phase at a flow rate of 0.8 mL/min.

### In vitro release kinetic testing

Free DFO, HA (215 kDa)-DFO and HA (752 kDa)-DFO conjugates were added to 4 mL of PBS (pH 7.4, 10 mM) at 37 °C and stirred at 5 × g in order to simulate a physiologic environment in vitro. The solutions of free DFO or HA-DFO conjugates were transferred into a dialysis bag (SnakeSkinTM, MWC0: 10 kDa) (Thermo Scientific Inc., Rockford, IL). Release medium was replaced with fresh PBS every 4 h to maintain the sink condition. A 60 µL solution was sampled and mixed with 3 mM FeCl (1:1 v/v) at predetermined intervals. DFO retention was quantified spectrophotometrically. Briefly, 3 mM of iron (III) chloride (FeCl_3_) was incubated with a series of standard solutions of DFO (0.05–0.5 mg/ml), 1 mg/ml HA (215 kDa)-DFO or HA (752 kDa)-DFO at ambient temperature for 16 h, followed by recording the absorption at 430 nm (DFO-Iron III chelate complex, ferrioxamine, maximum absorption), using a microplate reader (SpectraMax Gemini; Molecular Devices, Sunnyvale, CA).

### Cellular radiation protocol

Human Umbilical Vein Endothelial Cells (HUVECs) were obtained from Cell Applications, Inc. (San Diego, CA). Cells were grown in Cell Applications’ proprietary Endothelial Cell Growth Medium. All cells were utilized at passage two through four.

HUVECs near confluence were radiated using a Philips RT250 orthovoltage unit (250 kV X-rays, 15 mA; Kimtron Medical, Woodbury, CT), which delivers ionizing radiation through a filtered system. The cells were exposed to a dose of 5 Gy of radiation in a single fraction according to previously established protocols proven to successfully impair the growth and development of cellular cultures.

### Tubule formation assay

Matrigel (BD Biosciences, Franklin Lakes, NJ) was thawed and placed in eight-well chamber slides at 37 °C for 30 min to allow for solidification. Then, control, radiated, or DFO-radiated HUVEC cells (48,000 cells per well) were plated on Matrigel with 200 µL of 25% HUVEC proprietary media and 75% RMPI 1640 media. Cells were then incubated at 37 °C under 23% oxygen for 4 h. Variable formulations and doses of DFO were administered as follows: 50 µM DFO, 50 µM HA-DFO, 100 µM HA-DFO. All DFO was administered at the time of incubation. Cultures were recorded in real time with live cell imaging and photographed every hour for the duration of the experiment. Tubule formation was defined as a structure exhibiting a length four times its width and was analyzed in 10 random fields per well using an inverted Leica DMIL light microscope (Leica Microsystems, Wetzlar, Germany) at 100x magnification. Experiments were performed with a sample size of *n* = 6, and all data was quantified by three blinded, independent reviewers.

### In vivo study design and animal use

All animal experimentation was conducted in accordance with the guidelines published in the *Guide for the Care and Use of Laboratory Animals: Eighth Edition*.^45^ Protocols were approved by the University of Michigan’s Committee for the Utilization and Care of Animals (UCUCA) prior to implementation. Twelve-week-old male Sprague Dawley rats (*n* = 46) weighing approximately 400 g were acclimated for 7 days in light and temperature-controlled facilities and given food and water ad libitum. Rats were divided into four groups: Fx (control fracture, *n* = 5), XFx (irradiated fracture, *n* = 15), iDFO (irradiated fracture + injected deferoxamine, *n* = 15) and HA-DFO (irradiated fracture + hyaluronic acid-deferoxamine implanted at the time of surgery, *n* = 11). On day seven, rats underwent fractionated radiotherapy over a 5 day-period followed by a recuperation period of 14 days prior to surgery. During recovery, animals were acclimated to a soft chow high-calorie diet (Hills-Columbus Serum; Columbus, Ohio) to ensure adequate food intake and nutrition in the post-radiation and post-operative periods. Subsequently, animals underwent osteotomy, DFO injection/implantation and a 40-day consolidation period as outlined prior to dissection, bony union analysis, µCT imaging and biomechanical testing. All 46 animals completed the study, however, two animals in the XFx group had severely comminuted mandibles which precluded subsequent imaging and biomechanical testing. They were non-unions and thus still considered in the bony union analysis. Additionally, we powered the experiment based on expected magnitude of effect of the variables in question given our previous experience working with this radiated fracture model, so that the groups were not equal at the start of the experiment, as reported. Each measurement was taken from each distinct sample.

### Animal radiation delivery protocol

All radiation procedures were conducted in the Irradiation Core at the University of Michigan Cancer Center. After transient induction of anesthesia with an oxygen/isoflurane mixture, left hemi-mandibles were radiated using the Philips RT250 orthovoltage unit. Our selected region of interest (ROI) spans a 2 mm distance posterior to the third molar and correlates to the future site of surgically created osteotomy. Lead shielding is provided to ensure localized delivery and protection of surrounding tissues. A previously described Human Equivalent Dose of Radiation (HEDR) developed with the guidance of the department of radiation oncology at the University of Michigan was utilized.^46–48^ Briefly, a fractionated dose of 7 Gy per day was administered over 5 days for a total of 35 Gy. This is comparable to 70 Gy in human mandibular high-dose radiotherapy. This dose was designed to predictably replicate pathologies analogous to those observed in the setting of clinically advanced mandibular osteoradionecrosis, while taking the diminutive size of the mandible and surrounding tissues into consideration.

### DFO administration

The dose and delivery method of iDFO used in these protocols was derived from an extensive literature search regarding its use in long bone mouse models and the subsequent advancement of our experimentation with this drug over recent years.^22–29^ We have modified the reported dose to accommodate the larger volume of the rat mandible in our animal model. DFO (200 µM in 300 µL NS) was given as a local injection directly into the fracture site every other day starting on post-operative day 4 and continuing through post-operative day 12. This time frame was chosen to correlate with the reasonable time for initiation of angiogenesis in a murine fracture model.^8–10^ HA-DFO was implanted at the osteotomy site along the severed bone edges at the time of surgery. The dose of DFO loaded on the HA scaffold for delivery was calculated at 1000 µM which is equivalent to the 5 injections of DFO (200 µM each injection) given in our standard localized injection method.

### Peri-operative care

Gentamycin (30 mg/kg SQ) was given prophylactically before surgery and twice post-operatively. To ensure adequate analgesia, hydration and anesthesia, rats were given buprenorphine (0.15 mg/kg SQ) along with Lactated Ringers Solution (25 cc/kg SQ), and then anesthetized using an inhalational isoflurane/oxygen mixture throughout the surgical procedure. Post-operatively, animals were placed on warming blankets and monitored for heart and respiratory rates. Post-operative analgesia with buprenorphine was continued twice daily until POD 4, and as needed thereafter. Weight gain, porphyrin staining, food and fluid intake were assessed to determine the need for continued analgesia.

### Osteotomy and fixator placement

After standard preparation and draping a 2 cm midline incision was placed ventrally from the anterior submentum to the neck crease. After drilling a 1.1 mm hole a 1.5” #0–80 stainless steel rod was threaded horizontally across the anterior mandible and both ends brought externally through the skin, creating the anterior portion of our modified external fixator device. Bilateral holes were then drilled 2 mm anterior and superior to the mandibular angle, a #0–80 threaded pin was secured through a washer and brought externally through the skin for the posterior fixator placement. After stabilization of the fixator, a vertical osteotomy was created using a10 mm micro reciprocating blade (Stryker) ~2 mm anterior to the posterior washer on the left hemi-mandible, extending from the inferior margin of the mandible superiorly to the sigmoid notch along the anterior aspect of the coronoid process. After reduction of the osteotomy edges, hemostasis was verified, and incisions were closed in layers. Four hours after osteotomy, the fixator device was set to a 2 mm fixed distance for the length of the experiment as previously described.^49^

### Micro-CT imaging

Micro-CT (GE Healthcare Biosciences) images were obtained using 80 kVp, 80 mA and 1100 ms exposure at a resolution of 45-micron voxel size for bone analysis. The individual scans were reconstructed and reoriented in a three-dimensional x, y and z plane, then several rotations and cropping of non-bone space were undertaken for uniformity. The region of interest was selected correlating to the region of fracture healing, 2 mm directly behind the third molar. Using Microview software the ROI was then highlighted and analyzed for bone mineral density (BMD), tissue mineral density (TMD) and bone volume fraction (BVF).

### Biomechanical testing

Pins were inserted perpendicularly through the hemi-mandibles at both existing anterior and posterior pin-site holes. The posterior and anterior aspects of the mandible were potted using a Cerrobend Bismuth alloy. The mandible was positioned inside the pot with the inferior edge of the mandible perpendicular to the top edge of the pot. Using a servohydraulic-testing machine (858 Mini Bionics II; MTS Systems), potted hemi-mandibles were loaded to failure using tension at a constant displacement rate of 0.5 mm/s. Displacement was monitored with an external linear variable differential transducer (LVDT; Howard A. Schaevitz Technologies). The load and displacement data were acquired using the TestStar IIs system (version 2.4; MTS) at a sampling frequency of 200 Hz. A custom LabVIEW script was used to analyze the raw force-displacement data. Load-Displacement curves were analyzed for stiffness (S), and failure load (FL).

### Bony union analysis

En-bloc dissection of mandibles allowed for assessment of bony union. This was clinically defined as the absence of movement across the regenerate site on manual manipulation of the regenerate gap after removal of the fixator device. Union status was subsequently verified with micro-computed tomography (μCT) imaging where possible.

### Statistical analysis

All statistical analysis was conducted using SPSS software (v24; IBM). Outcome metrics were compared utilizing a one-way analysis of variance and reported as means ± standard deviation with *p* *<* 0.05 considered statistically significant. Points of significance are symbolized graphically (*). No covariates, assumptions or corrections were employed. A post hoc Tukey test was used for multiple comparisons.

### Reporting summary

Further information on research design is available in the Nature Research Reporting Summary linked to this article.

## Supplementary information

Supplementary Information

Supplementary Information Movie 1

Reporting Summary

## Data Availability

All supporting data in the published article generated during the study can be obtained by contacting the corresponding author.

## Supplementary information

**Supplementary information** accompanies the paper on the *npj Regenerative Medicine* website (10.1038/s41536-019-0072-9).
