# Supplementary material for: Implantable hyaluronic acid-deferoxamine conjugate prevents nonunions through stimulation of neovascularization
Source: NPJ Regen Med. 2019 May 21;4:11. doi: 10.1038/s41536-019-0072-9 (PMC6529413; doi:10.1038/s41536-019-0072-9)
Supplement: Supplementary file 1 — Supplementary Information [file 41536_2019_72_MOESM1_ESM.pdf]

Supplementary Information (List)

Fig. S1: Still Image for Movie S1 and Caption

Movie S1

-Fig. S1-Still Image for Movie S1 and Caption.

HA-DFO stimulates angiogenesis in vitro in HUVEC cells exposed to radiation. Three groups of irradiated HUVECs demonstrate variable tubule formation in response to deferoxamine despite radiation injury. Note the increased tubule density and organization in the 100µM HA-DFO sample when compared to 50µM DFO and 50µM HA-DFO at 2h of incubation.

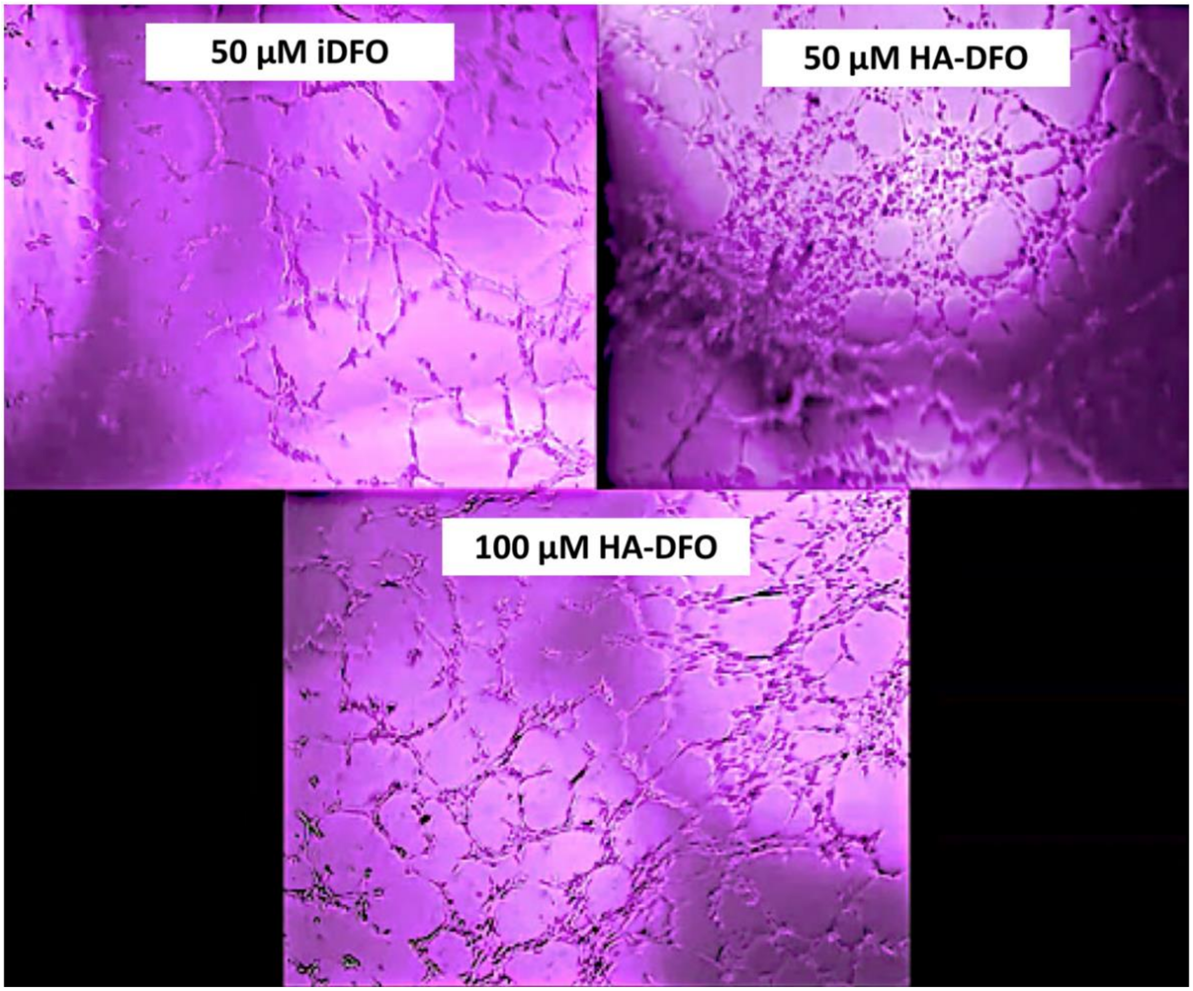

-Movie S1

Live cell imaging video of HUVEC cells exposed to radiation and angiogenic response to treatments despite radiation exposure. (See separate Movie S1 file)
